# Objective and subjective assessment of back shape and function in persons with and without low back pain

**DOI:** 10.1038/s41598-025-03901-z

**Published:** 2025-06-20

**Authors:** N. Taheri, L. Becker, L. Fleig, F. Schömig, B. U. Hoehl, L. M. S. Cordes, U. Grittner, L. Mödl, H. Schmidt, M. Pumberger

**Affiliations:** 1 Center for Musculoskeletal Surgery, Charité – Universitätsmedizin Berlin, Corporate Member of Freie Universität Berlin, Humboldt-Universität Zu Berlin, Luisenstraße 64, 10117 Berlin, Germany; 2 Berlin Institute of Health, Julius Wolff Institute for Biomechanics and Musculoskeletal Regeneration, Charité-Universitätsmedizin Berlin, Berlin, Germany; 3 Department of Psychology, MSB Medical School Berlin, Berlin, Germany; 4 Charité – Universitätsmedizin Berlin, Corporate Member of Freie Universität Berlin and Humboldt-Universität Zu Berlin, Institute of Biometry and Clinical Epidemiology, Charitéplatz 1, 10117 Berlin, Germany

**Keywords:** Low back pain, LBP, Chronic low back pain, cLBP, Self-reported impairment of back shape and function, Chronic pain, Musculoskeletal abnormalities

## Abstract

Individuals with chronic low back pain (cLBP) may self-report about impairment of their back shape and function. As classical clinical diagnostic modalities seem to provide limited information on the pathogenesis of cLBP, interest has shifted to a more comprehensive approach of diagnosing cLBP. Self-reported outcome measurements in the form of either questionnaires or as part of clinical interview have gained interest. In theory, these self-reported assessments on one’s LBP provide the clinician with substantial information regarding the dominance of specific factors in a rather complex bio-psycho-social interplay of factors leading to cLBP. In order to analyze how well self-reported impairment (SRI) corresponds with objective measures, we evaluated the association between SRI and objectively measured back shape and function. In a cross-sectional study, we included 914 participants (207 asymptomatic, 480 non-chronic LBP (ncLBP), 227 cLBP). Participants were categorized into three groups: asymptomatic participants did not report back pain. Participants with back pain lasting for 12 weeks or more were categorized as cLBP patients, while participants with back pain for less than 12 weeks were classified as non-chronic LBP patients (ncLBP). Back function was quantified using finger-to-floor distance (FFD), Ott and Schober test, and 30 s sit-to-stand test (STS). Back shape and function were measured in standing position using a computer-assisted medical device. SRI was quantified during a clinical interview using a numerical 10–score-scale (1: unrestricted, 10: severely restricted). Higher SRI was associated with worse performance in every clinical test. Effect estimates ranged from small (Ott test: β = −0.05, CI −0.09–0.00, η^2^ = 0.01; p = 0.05; Schober test: β = 0.08, CI −0.13 −0.04, η^2^ = 0.01, p < 0.01) to moderate (FFD: β = 1.66, CI 1.27–2.19, η^2^ = 0.05, p = 0.05; STS: β = −0.08, CI −0.82, CI −1.06–−0.59, η^2^ = p < 0.01) in participants with ncLBP and cLBP. Higher SRI was associated with pathological back shape (hyperkyphosis, β = −0.03, CI = −0.29–0.51, η^2^ = 0.01; p = 0.58 and hyperlordosis, β = 0.35, CI 0.04–0.65, η^2^ = 0.02, p = 0.03) as well as attenuation of range of motion in the frontal and sagittal planes in every direction except for the thoracic range of extension. Effect sizes were small (η^2^ = 0.01–0.04). This study demonstrated an association of SRI with objective back shape and function. Participants with ncLBP seem to have the highest correspondence between objective evaluation and SRI of back shape an function. In the future, these associations can be used to further personalize both diagnostic and therapeutic modalities for individuals suffering from LBP rather than generalizing treatment options.

## Introduction

Low back pain (LBP) is the leading cause of years lived with disability and has a lifetime prevalence of 80%, contributing to high rates of work absence, hospital admissions, and significant impacts on quality of life and productivity. These effects result in substantial direct and indirect costs for healthcare systems worldwide^1,2^. Chronic low back pain (cLBP), affects 4–25% of individuals depending on age and sex^3^. cLBP patients often suffer persistent pain and discomfort, leading to marked impairments in physical function and daily activities. In addition to physical limitations, cLBP has significant psychological and social effects, including anxiety, depression, and reduced occupational productivity. The physical disabilities caused by cLBP, such as diminished mobility, strength, and coordination, hinder routine tasks like lifting, bending, and walking. The chronic nature of the pain can intensify emotional distress, which compounds the patient’s overall disability^1,3,4^.

Clinical assessment of back shape and function is essential in cLBP patients [Wong 2004; Kowalski 2022]. Physical examinations commonly include basic range of motion (RoM) measurements and specific tests such as the finger-to-floor distance (FFD)^5,6^, Schober^7^, Ott test, and the 30-s sit-to-stand test (STS)^8,9^.

Functional assessments help clinicians evaluate how cLBP affects a patient’s ability to perform daily activities, work tasks, and recreational pursuits. These assessments also provide objective data on mobility, strength, flexibility, and endurance, identifying specific impairments that guide personalized treatment and rehabilitation strategies^10,11^. They facilitate communication between healthcare providers and patients, setting realistic recovery goals and expectations. These evaluations contribute to research by offering standardized metrics to assess the effectiveness of various therapeutic interventions, helping to shape evidence-based practice guidelines^12–14^.

Studies suggest that cLBP patients tend to report worse spinal function compared to non-chronic LBP (ncLBP) patients and asymptomatic individuals^15^. Self-reported back function in cLBP patients may be influenced by selective attention focused on pain, potentially exacerbating functional impairments^16^. Emotional responses to pain can lead to avoidance behaviors, further degrading back function^17^. Research by Liegl et al. has demonstrated that self-reported impairment (SRI) often align with objective physical performance measures^18^. This is especially important to know for clinicians as studies have shown that health care professionals can be biased in their assessment and treatment decisions (i.e. bias towards unimodal, somatic assessments for LBP patient^19^. Often, clinicians still adhere to a biomedical model rather than acknowledging the importance of a biopsychosocial model disregarding the potentially significance of self-reported disability and psychosocial factors^19–21^. This can further weaken trust within LBP patients and ameliorate the outcome of therapeutic interventions^22^. An extensive review of current LBP guidelines has summarized that psychosocial factors are underestimated within the guidelines ^23^. This study aimed to explore how changes in self-reported back shape and function correlate with objective measurements of back function.By providing further evidence of an association between SRI and actual objective changes, this can help the diagnostic and therapeutic process. This is of the utmost importance, as SRI in LBP patients has been linked to both coherence to therapeutic modalities as well as the resulting disability^24^.

We hypothesize that participants with both ncLBP and cLBP will report greater impairments in back shape and function than asymptomatic individuals. Additionally, we expect that higher SRI will be associated with objective declines in back shape and function. Finally, we examined whether these associations varied between individuals reporting no pain, chronic pain, or no chronic pain.

## Materials & methods

### Ethics approval

This study was approved by the local ethics committee of Charité – Universitätsmedizin Berlin (EA4/011/10). Written informed consent was obtained from all study participants. The presented study is the result of a prospective cross-sectional study to characterize the changes in biomechanics, psychosocial risk factors as well as structural abnormalities and their interaction in cLBP. The study has been pre-registered at the German national trial registry (DRKS00027907XXX). Furthermore, the study was conducted in accordance to the STROBE guidelines for observational studies as well as in accordance with the declaration of Helsinki.

### Recruitment, in - & exclusion criteria

We included participants the minimum age of 18 and the maximum age of 65 years were included in this study. Knowledge of the German language was needed to be eligible. Participants were excluded if they suffered from neurological movement disorders, acute radiculopathy, systemic diseases requiring medication, or malignant diseases. Furthermore, a BMI > 28 kg/m^2^ was an exclusion criterion due to the usage of non-invasive measurement devices for spinal shape, which show an excellent agreement below this threshold but insufficient agreement in overweight persons^25^. The recruitment of study participants employed a multifaceted approach to ensure comprehensive outreach and enrollment. Initial efforts involved collaboration with local healthcare providers and clinics specializing in spine disorders, where potential participants were identified based on diagnostic criteria and suitability for the study. Additionally, partnerships were established with local companies, leveraging their networks to reach individuals affected by LBP within the working-age population. A strategic media campaign was conducted to enhance visibility, utilizing press releases, television appearances, and targeted advertising to raise awareness about the study and encourage participation. Flyer distribution in community centers, health fairs, and medical facilities supplemented these efforts, providing accessible information to interested individuals. Furthermore, word-of-mouth referrals played a crucial role, with participants encouraged to share study details with peers and acquaintances. Throughout the recruitment process, stringent ethical guidelines were followed, including informed consent procedures and protection of participant confidentiality. In order to reduce potential bias, we formulated clear inclusion and exclusion criteria, ensured recruitment from diverse sources, especially socio-economically and geographically, to avoid location-based bias. Furthermore, cultural and language sensitivity in the recruitment process were strictly adhered to in order to ensure that participants thoroughly understand the questionnaires and clinical examination.

### Definiton of cLBP and ncLBP

Participants that did not report any back pain during the measurements were characterized as asymptomatic. Participants who had suffered from back pain of the lumbar spine on nearly every day for at least 12 weeks were considered participants with cLBP and participants who had suffered from LBP for less than 12 weeks and/or in the form of intermitting pain periods were considered as participants with ncLBP.

### Measurement protocol

Participants were asked to answer the following standardized and validated participant-reported outcome measurements (PROMs). Furthermore, participants underwent clinical examination by a resident of orthopaedic surgery with several years of clinical experience. The standardized examination included evaluation of acute symptoms and medical history as well as thorough analysis of back, hip and hamstring mobility. Lastly, participants back shape and function was analyzed by a health professional using a non-invasive device.

### Self-reported assessment of back shape and function

During the clinical examination, all study participants were asked to rate their back shape function on a scale of 1–10. 1 describes no subjective restriction of back function, while 10 represents severe restriction of spinal function.

### Objective functional assessments

#### Ott test

The Ott test is used to assess the mobility of the thoracic spine. With the participant in upright standing position, the examiner marks the spinous process of the seventh cervical vertebra and a point 30 cm below. The patient is then asked to bend forward as far as possible while the examiner measures the increase in distance between the two marked points.

#### Schober test

The Schober test is used to measure the flexion of the lumbar spine. During the test, the patient stands with their feet shoulder-width apart and the examiner marks the skin in between the posterior superior iliac spine, which corresponds fairly to S1 and a point 10 cm above. The patient is then asked to bend forward as far as possible while the examiner measures the distance between the two marked points.

#### Finger-floor-distance (FFD) test

The FFD test is used to assess the flexibility of the spino-pelvic mobility as well as the hamstrings. During the test, the patient stands with their feet together and reaches forward with their hands towards the floor. The examiner measures the distance between the fingertips and the floor.

#### Sit-to-stand (STS) test

The STS test is a clinical tool used to assess lower extremity strength. During the test, the patient sits on a chair and is asked to stand up and sit down as many times as possible in 30 s.

#### Back mobility in flexion, extension, and lateral bending

The Idiag M360® (MediMouse, Idiag AG, Fehraltorf, Switzerland) was used to evaluate back shape and function. The device is rolled from the spinous process of the seventh cervical vertebra along the spinous processes to the anal fold. For reproducible measurements, the spinous process of the seventh cervical vertebra and a reference point 2 cm below the junction of the left and right posterior superior iliac spines are marked. The validity and reliability have been established in previous studies^25–28^. Measurements were performed in upright standing as well as in left and right lateral bending, flexion, and extension. Each posture was measured three times.

### Statistical analysis

To evaluate the association between the SRI(independent variable), back shape and function parameters (dependent variables, i.e., FFD, Ott, Schober, STS, thoracic Cobb, thoracic bending to the left and right, lumbar cobb, lumbar bending to the left and right, thoracic kyphosis, thoracic extension and flexion, lumbar lordosis, lumbar extension and flexion), we used two separate linear regression models for each dependent variable. Both models included a nominal covariate for the respective groups (asymptomatic, cLBP, ncLBP) adjusted for age, sex (male, female) and body mass index (BMI). The analysis was divided into two models. Model A evaluated the main effects between the back pain groups and SA. Model B analyzed and evaluated the interaction between the group and SA. Furthermore, Model B tested different associations between SA and back functioning parameters. Model Parameters of Model B were only reported if at least small interaction effects of differential association between SA and functional or shape parameters were present.

The estimated mean differences between asymptomatic participants and the respective pain groups are reported together with 95% confidence intervals (CIs) and partial η^2^ ‘s as a measure of effect size. A partial η^2^ < 0.01 was considered a very small effect, a partial 0.01 ≤ η^2^ < 0.06 a small effect, a partial 0.06 ≤ η^2^ < 0.14 a moderate effect, and a partial η^2^ ≥ 0.14 a large effect. The interpretation of effect sizes was based on scientific evidence^29,30^. Furthermore, p-values were examined for demographics and both linear regression models. A two-sided-significance level of 0.05 was used. Yet, the interpretation of p-values has to be performed with caution, as p-values are no effect sizes, are dependent on sampe size. Additionaly interpretation of p-values is difficult in situation of multiple testing without any adjustment for multiple test^31,32^. Additionaly interpretation of p-values is difficult in the situation of multiple testing without any adjustment for multiple testing^31,32^. R version 4.3.1 and SPSS version 27.0 were used for data analysis.

## Results

### Demographics

914 participants were included in this analysis. The average age was 41.9 years (Standard deviation, SD = 12.4). 207 participants were asymptomatic, 480 participants are suffering from ncLBP and 227 were suffering from cLBP. Groups were similar regarding the sex distribution or BMI (Table 1). Participants suffering from cLBP were on average 3 to 4 years older than the other participants.

**Table 1:** Demographics of the total participants; cLBP, ncLBP and asymptomatic participants.

	Total Mean ± SD (n = 914)	cLBP Mean ± SD (n = 227)	ncLBP Mean ± SD (n = 480)	Asymp Mean ± SD (n = 207)
Female	493	123	260	110
Male	421	104	220	97
**BMI [kg/m**^**2**^**]**	23.66 ± 2.97	23.66 ± 2.85	23.56 ± 3.1	23.87 ± 2.89
Age [years]	41.93 ± 12.4	44.45 ± 11.73	41.28 ± 12.41	40.18 ± 12.92
Occupational status:				
Employed	892	226	473	203
Unemployed	22	11	7	4
Marital status:				
Married	619	154	179	119
Single	295	73	301	88

*cLBP* chronic low back pain, *ncLBP* non-chronic low back pain, *SD* standard deviation.

Asymptomatic participants had a mean SRI of 2.6 (± 1.5), while ncLBP participants presented mean values of 2.9 (± 1.6) and cLBP patients showed the worse SRI with values of 4.3 (± 1.9) (Table 2).

**Table 2:** Descriptive statistics for the variables under study presented by group; *asymp* asymptomatic (n = 207), *cLBP* chronic low back pain (n = 227), *ncLBP* non-chronic low back pain (n = 480), *FFD* finger-floor-distance, *STS* sit-to-stand test.

	Asymp. n = 207 Mean ± SD	ncLBP n = 480 Mean ± SD	cLBP n = 227 Mean ± SD
Self-reported impairment	2.6 ± 1.5	2.9 ± 1.6	4.3 ± 1.9
Back shape			
Thoracic cobb	−2.4 ± 6.2°	3.0 ± 6.5°	4.2 ± 6.4°
Lumbar cobb	2.3 ± 5.2°	2.4 ± 5.5°	3.4 ± 5.1°
Thoracic kyphosis	43.5 ± 1.0°	43.4 ± 10.6°	44.8 ± 10.3°
Lumbar lordosis	25.7 ± 7.4°	24.4 ± 8.3°	24.5 ± 8.4°
Back function			
Ott test	2.6 ± 1.1	2.6 ± 1.2	2.4 ± 1.0
Schober test	4.8 ± 1.2	4.6 ± 1.2	4.3 ± 1.3
FFD	1.0 ± 11.9	2.7 ± 11.5	5.0 ± 13.4
STS	24.0 ± 6.0	22.9 ± 6.1	21.0 ± 5.5
Thoracic bending left	39.3 ± 11.9°	39.8 ± 11.3°	38.8 ± 12.5°
Thoracic bending right	37.1 ± 10.1°	36.5 ± 9.7°	36.2 ± 10.4°
Thoracic extension	15.1 ± 10.2°	13.8 ± 9.4°	16.1 ± 10.0
Thoracic flexion	23.1 ± 11.1°	24.5 ± 11.7°	24.5 ± 11.0°
Lumbar bending left	16.0 ± 7.2°	16.2 ± 7.2°	15.4 ± 8.0°
Lumbar bending right	17.8 ± 7.2°	17.4 ± 7.3°	16.6 ± 7.9°
Lumbar extension	9.3 ± 6.2°	9.4 ± 6.3°	7.4 ± 5.7°
Lumbar flexion	49.4 ± 9.8°	47.0 ± 9.5°	45.9 ± 11.4°

### Ott test, Schober test, STS & FFD

Higher impairment of self-reported back shape and function were associated with worse performance in all clinical tests. The effect size of the associations was very small for Ott test, small for Schober and STS tests, and moderate for FFD (Panel A in Table 3). Further analysis revealed worse performance in Schober test and FFD for the all the groups. With worsening self-reported back function, all three groups performed worse in the test. Effect size of interactions was small (Panel B in Table 3).

**Table 3:** Estimated average differences in Ott, Schober, STS and FFD for a one-point higher impairment of self-reported back shape and function for each backpain group; Panel A: model with main effects (same association in each group); Panel B: models with interactions between FAB and group; only reported in case of effects (partial η^2^ ≥ 0.01).

Score (independent variable)	Parameter (dependent variable)	A (main effect)						B (marginal effects for interaction)				
		Estimate	95% CI		Partial η2	p-value		Estimate	95% CI		Partial η2	p-value
Subjective back function	Ott	−0.05	−0.09	0.00	0.00	0.05	Asymp					
							cLBP					
							ncLBP					
	Schober	−0.08	−0.13	−0.04	0.01	< 0.01	Asymp	−0.13	−0.22	−0.05	0.01	0.01
							cLBP	−0.13	−0.22	−0.05		0.36
							ncLBP	−0.17	−0.29	−0.05		< 0.01
	STS	−0.82	−1.06	−0.59	0.06	< 0.01	Asymp	−1.30	−1.88	−0.73	0.00	< 0.01
							cLBP	−0.77	−1.08	−0.45		< 0.01
							ncLBP	−0.68	−199	−0.27		< 0.01
	FFD	1.66	1.19	2.13	0.05	< 0.01	Asymp	2.3	1.12	3.47	0.01	< 0.01
							cLBP	2.17	1.34	3.01		
							ncLBP	1.18	1.34	1.82		

*asymp* asymptomatic (n = 207), *cLBP* chronic low back pain (n = 227), *ncLBP* non-chronic low back pain (n = 480).

### Back shape and mobility in flexion, extension, and lateral bending

Higher impairment of self-reported back shape and function were associated with lower thoracic Cobb angle, lumbar cobb angle thoracic range of bending, lumbar range of bending, thoracic flexion, lumbar extension and lumbar flexion. Higher SA scores were associated with higher thoracic kyphosis, lumbar lordosis and thoracic extension. Effect sizes were very small or small. (Panel A in Table 4). Higher values in self-reported back function were associated with hyperkyphosis in asymptomatic and ncLBP Patients and hypokyphosis in cLBP patients. Furthermore, lumbar lordosis decreased in asymptomatic participants and increased in cLBP and ncLBP patients in association with self-reported back function. There were relevant interactions for thoracic & lumbar bending to the left, thoracic kyphosis, lumbar lordosis and lumbar flexion (Panel B in Table 4). Higher values in self-reported back function were associated to higher range of thoracic bending to the left as well as lumbar bending to the left in asymptomatic individuals and to lower range of bending in both cLBP and ncLBP patients. In association with higher self-reported impairment of back shape and function, lumbar range of flexion worsened in every analyzed group. Effect sizes were small.

**Table 4:** Estimated average differences of non-invasive device measurements for a one-point higher impairment of self-reported back shape and function for each back-pain group; Panel A: model with main effects (same association in each group); Panel B: models with interactions between FAB and group; only reported in case of effects (partial η^2^ ≥ 0.01).

Score (independent variable)	Parameter (dependent variable)	A (main effect)						B (marginal effects for interaction)				
		Estimate	95% CI		Partial η2	p-value		Estimate	95% CI		Partial η2	p-value
Subjective SA	Thoracic cobb	−0.03	−0.29	0.23	0.00	0.82	Asymp					
							cLBP					
							ncLBP					
	Thoracic bending left	−0.37	−0.85	0.11	0.00	0.13	Asymp					
							cLBP					
							ncLBP					
	Thoracic bending right	−0.38	−0.79	0.03	0.0	0.07	Asymp					
							cLBP					
							ncLBP					
	Lumbar cobb	−0.02	−0.24	0.20	0.00	0.85	Asymp					
							cLBP					
							ncLBP					
	Lumbar	−0.34	−0.64	− 0.05	0.01	0.02	Asymp	0.27	0.46	1.00	0.00	0.47

*asymp* asymptomatic (n = 207), *cLBP* chronic low back pain (n = 227), *ncLBP* non-chronic low back pain (n = 480).

## Discussion

This study showed small to moderate effect size between worse SRI of back function and alterations of back shape (hyperkyphosis, hyperlordosis), and back function, in both cLBP and ncLBP patients. The results showed that in both LBP groups, worse SRI was associated with more pathological back shape and reduced range of motion.

Self-reported changes of an individual’s perception have been implied to be highly important in the multidimensional etiology, chronification, and resulting disability of LBP^33–35^. In considering established clinical examinations for evaluating and monitoring lumbar and hamstring mobility, we found a small to moderate association between SRI and back shape and function in ncLBP and cLBP patients. The small to moderate association of SRI with STS and FFD is a clinically relevant finding. Stressful situations, and low self-esteem, and altered self-perception of the individual’s general health and functional status may consecutively lead to higher perceived disability, which has been shown to manifest in reduced hamstring mobility^33^. Additionally, low self-esteem and stress lead to an increased cortisol secretion, which elevates sympathetic nervous system activity. The resulting increase in sympathetic activity causes heightened muscle tension, further impair mobility^36–39^. Lastly, negative perception of impaired back shape and function could result in a stress response that may induce further muscle atrophy, as demonstrated in mouse models^40,41^.

Furthermore, there are common misbeliefs from clinician’s point of view when treating patients with LBP in both an out – and in-patient setting. These misconceptions often stem from a misunderstanding of the etiology of LBP as clinicians diagnose and treat LBP from a biomedical model rather than recognizing psychosocial risk factors^20–22^. This leads to poor indication of diagnostic modalities^42,43^ and potentially mitigate of inappropriate treatments options, such as inappropriate surgical treatment for lumbar disc herniation that ultimately did not cause or contribute to LBP^44^. A large data base study, has shown that 75% of lumbar spine MRI’s conducted in the central Denmark region as referrals from primary care were inappropriate^45^. This may potentially lead to patient’ dissatisfaction and further aggravate the pain-related disability^46,47^. Creating an environment of active listening to patients’ beliefs and their SRI as part of an interdisciplinary, multimodal diagnostic approach^19^, could potentially increase coherence with therapeutic modalities^24^.The small and moderate effect sizes in our study warrant careful interpretation. The strength of the associated measures is limited, yet this does not diminish their clinical relevance. Small alteration in perception and function can have significant consequences and implications for individuals as prior research has shown these changes to further influence pain experiences and rehabilitation^33,34^. While larger effect sizes would strengthen our conclusions, the current findings still support the relevance of SRI in the bio-psycho-social model of LBP.

The discrepancy in SRI accuracy between the subgroups is one of the main findings of the study. LBP patients tended to report SRI consistent with objective measures, while asymptomatic individuals often showed inconsistent associations. This could potentially be due to intermittent pain episodes that influence the awareness of functional limitation^48^. The cognitive biases in forms of pain catastrophizing as well as fear-avoidance beliefs could potentially impact a heightened focus actual back shape and function in LBP patients^48,49^. In contrast, asymptomatic participants might rely on general well-being rather than objective mobility metrics^50,51^.

Considering the back shape measurements, the present study showed an associated impairment in the form of hyperkyphosis and hyperlordosis and a reduced range of motion in every movement direction except for thoracic extension. Further analysis revealed that the impairment of above-mentioned parameters associated with worse self-reported values was more severe in ncLBP than in cLBP patients. It remains unclear whether this finding is incidental or could be a result of chronification process. Notably, the moderate association of SA with lumbar flexion is particularly important. The range of lumbar flexion is highly relevant for participants suffering from LBP. Previous studies have suggested that the range of lumbar flexion is equivalent to good or bad back function^50,51^. This could suggest lumbar flexion as a potentially self-intuitive indicator of objective disability^51^.

Changes in movement – both in the frontal and sagittal planes – could possibly be due to altered muscle activation caused by changes in sympathicotonus^36–39^. Increased muscle tension can exacerbate muscular dysbalances, as reported in the literature, leading to asymmetric flexion–extension movements and reduced movement amplitude^39^. Attenuated multifidus contraction in cLBP has been shown to coexist with fear, depression and anxiety^52^.

In the frontal plane, only ncLBP patients showed significant differences during the examination of thoracic bending to the left, with small effect sizes. The correlation of the back measurements in the frontal plane with bending X-rays has been shown to be weak^53^], although other studies proved have shown its reliability in the frontal plane, despite a weak correlation^54^. For measurement with the Idiag M360® in the sagittal plane, several studies have shown excellent correlation between the device’s RoM and x-ray RoM^25–28^.

The findings emphasize the importance of SRI in LBP assessment and treatment. Encouraging active listening and incorporating SRI into clinical decision-making may improve patient adherence to therapeutic interventions^24^. Possible therapeutic approaches can implement psycho-educative strategies to further facilitate restoration of function^55–57^.

Certain limitations of this study needs to be discussed. First, the cross-sectional designs prevents any causal interferences. While we were able to observe association between SRI, back shape and function, we cannot determine whether poor SRI contributes to functional impairments of vice versa. Future longitudinal studies are needed to clarify these relationship.

Second, measurement errors may have contributed to biased results. While every examiner in the study was trained and initially supervised, individual movement execution and examiner judgement could introduce variability. Furthermore, subtle deviations of either muscle engagement or positioning can affect both the examiner’s as well as the patient’s accuracy.

Third, reliance on self-reported data carries the risk of response bias. While the effect of SRI on spinal load has been evaluated to some extent^35^, a quantification of actual impairment in terms of RoM has been lacking. Therefore, comparisons to other findings are not possible. Pain catastrophizing as well as fear-avoidance beliefs can influence participants perception of their SRI^35,49^. Lastly, the strict BMI cut-off of 28 kg/m^2^ could potentially introduce a bias towards a heathier population as obesity Is a known risk factor for LBP. Yet, it is also a risk factor for other major diseases and potentially worsens self-perception and therefore also SRI. While this criterion contributes to controlling of confounders, it can potentially limit the generalizability of our findings to the broader population. In summary, our results suggest that asymptomatic individuals tend to underestimate their back, while individuals with LBP – especially cLBP – seem to have closer association of their individual perception with objective measures between SRI and objective measures. Additionally, we observed that SRI is closely linked with worse back shape and function, particularly in the lumbar region. SRI showed the strongest association with lumbar flexion, potentially implicating the ability to flex the lumbar spine as a marker for LBP disability.

In order to determine whether changes of SRI correspond with changes of back shape and function over time, longitudinal studies are needed. Interventional studies using biofeedback techniques may provide valuable insight on the impact of positive feedback on SRI and ultimately back shape and function.

## Data Availability

The datasets generated and analyzed during the current study are available from the corresponding author on reasonable request.
